# Supplementary material for: Drosophila Clueless ribonucleoprotein particles display novel dynamics that rely on the availability of functional protein and polysome equilibrium
Source: bioRxiv. 2024 Aug 22:2024.08.21.609023. Preprint. [Version 1] doi: 10.1101/2024.08.21.609023 (PMC11370489; doi:10.1101/2024.08.21.609023)

Supplementary Fig 1. Cycloheximide decreases Processing bodies *ex vivo*

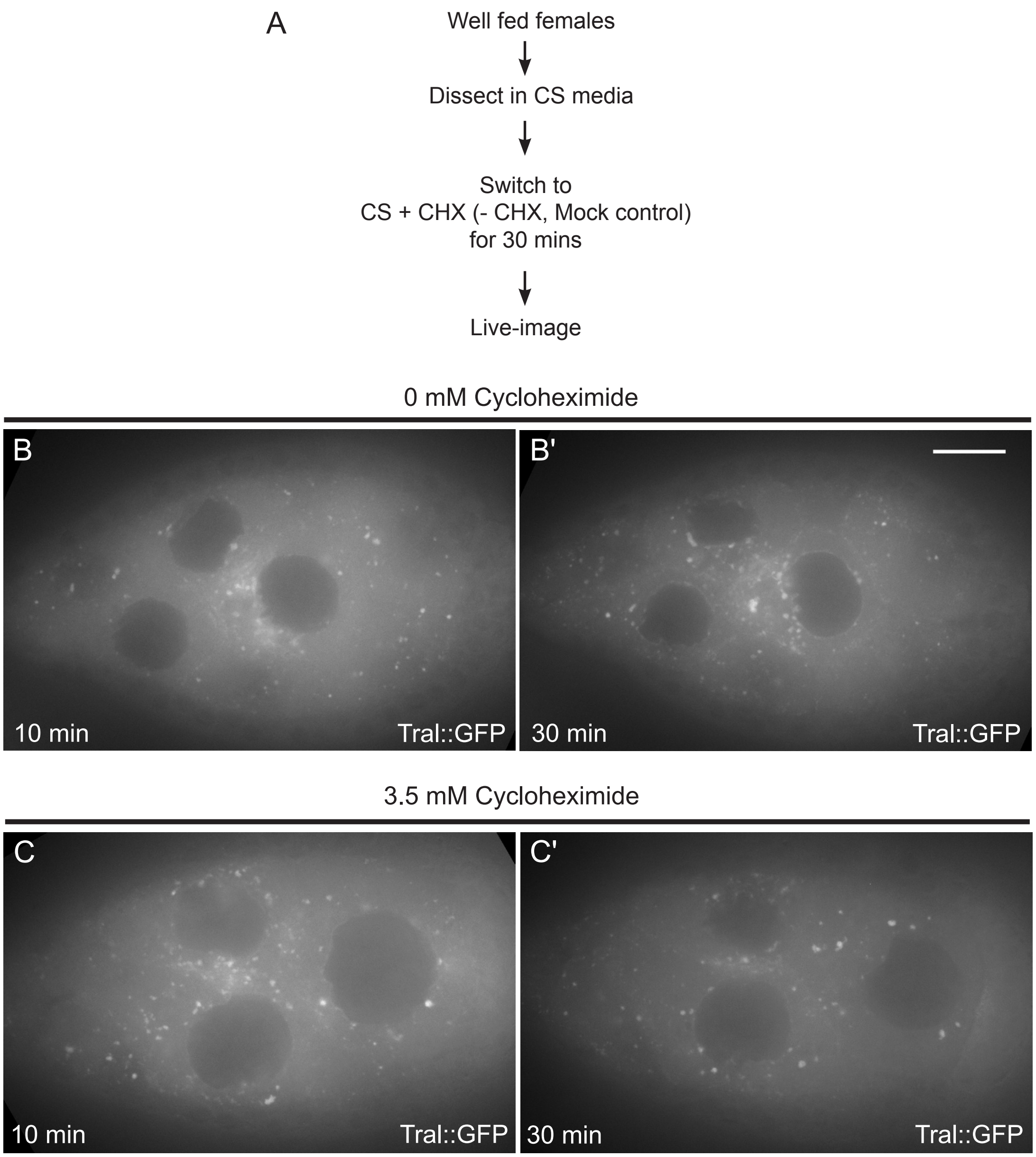

Supplementary Fig 2. Clu particles associated with mitochondria move more slowly

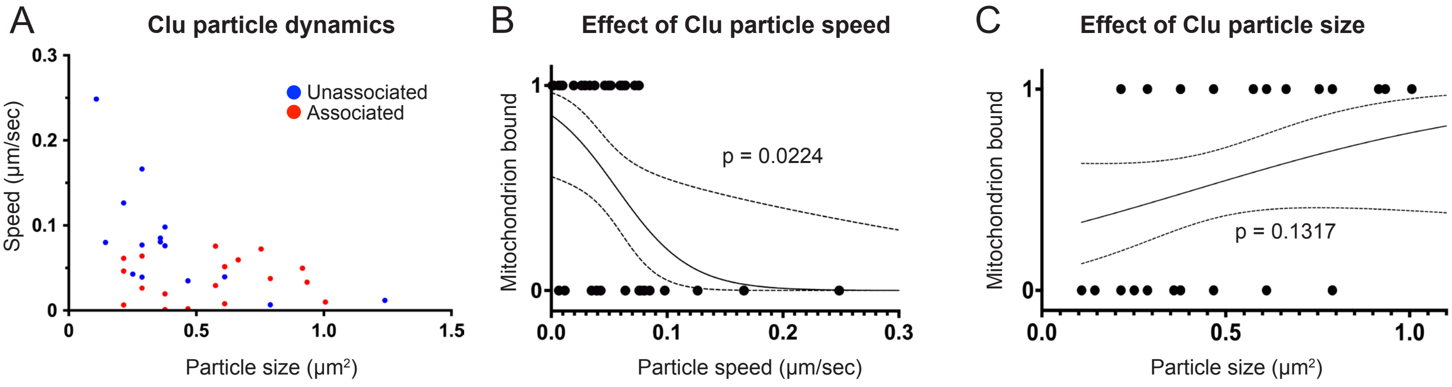

Supplement: Supplement 4 — S1 Fig. Cycloheximide causes reduced sizes and numbers of P-bodies ex vivo. (A) Workflow for the experiment. Well-fed tralCA06517 females were treated with 3.5 mM CHX for 30 minutes, and then live-imaged (B-C’). (B-B’) Still-image of stage 8 follicle showing a mock-control increases P-bodies. 68% of nurse cells had increased numbers and sizes of P-bodies in 30 minutes after mock treatment, and 32% of nurse cells had no changes (n=19 follicles, see S2 Table for details). (C-C’) Still-image of stage 8 follicle showing 3.5 mM CHX treatment decreases P-bodies. 48% of nurse cells had decreased numbers and sizes of P-bodies in 30 minutes after 3.5 mM CHX treatment, 43% of nurse cells had no changes, and 9 % of nurse cells had increased (n=23 follicles, see S2 Table for details). Images are 2 μm projections assembled from 0.5 μm sections. The focal plane was selected to have at least three to four nurse cells with a clear visibility of nuclear and cytoplasmic area, aiming for approximately 25% depth from the top surface of a follicle. Changes in P-bodies were determined by a subjective measurement. Follicle stages analyzed (n): mock control, stage 6 (7), stage 7 (10), stage 8 (2); 3.5 mM CHX, stage 5 (4), stage 6 (5), stage 7 (6), stage 8 (8). More details, including the number of follicles showing changes in the numbers/sizes of P-bodies by CHX treatment and the number of dissected animals, are described in S2 Table. Scale bar = 20 μm in B’ for B-C’. S2 Fig: Clu particle dynamics and mitochondria (A-C) Replicates of the experiment of Fig 9, analysis of Clu particle speed and mitochondrial association. (A) The graph plotted by speed, size, and mitochondrial binding of each Clu particle. Red represents the particles binding to mitochondria and blue represents the particles not binding to mitochondria. n=34. (B, C) Simple logistic regression analysis of (A). This predicts a probability of mitochondrial binding of the Clu particle depending on the particle speed (B) or size ( [file media-4.pdf]
