# Supplementary material for: Drosophila Clueless ribonucleoprotein particles display novel dynamics that rely on the availability of functional protein and polysome equilibrium
Source: bioRxiv. 2024 Aug 22:2024.08.21.609023. Preprint. [Version 1] doi: 10.1101/2024.08.21.609023 (PMC11370489; doi:10.1101/2024.08.21.609023)
Supplement: Supplement 18 [file NIHPP2024.08.21.609023v1-supplement-18.pdf]

bioRxiv preprint doi: https://doi.org/10.1101/2024.08.21.609023; this version posted August 22, 2024. The copyright holder for this preprint (which was not certified by peer review) is the author/funder. This article is a US Government work. It is not subject to copyright under 17 USC 105 and is also made available for use under a CC0 license.

Supporting information

Supplementary methods

Fly stocks

Fly stocks were maintained on standard cornmeal fly media. Animals were grown at room temperature. *w**; *P{PTT-GA} tral*^{CA06517} (Buszczak et al., 2007) were grown at room temperature. A newly eclosed fly is considered day 0.

bioRxiv preprint doi: https://doi.org/10.1101/2024.08.21.609023; this version posted August 22, 2024. The copyright holder for this preprint (which was not certified by peer review) is the author/funder. This article is a US Government work. It is not subject to copyright under 17 USC 105 and is also made available for use under a CC0 license.

Live-imaging for P-bodies

To test the CHX effect on disaggregation of P-bodies, ovaries were dissected from *w**; *P{PTT-GA} tral*^{CA06517} in CS, and then incubated for 30 minutes with CS containing 3.5 mM CHX (CS/CHX) following twice wash with the same media. Live images were obtained using a Nikon Eclipse Ti2 spinning disk microscope at 100x (Nikon Corporation, Tokyo, Japan). The follicles were mainly chosen in stages 7-8 that show more numbers of P-bodies than earlier stages. The focal plane was selected to have at least three to four nurse cells with a clear visibility of nuclear and cytoplasmic area, with approximately 25% depth from the top surface of a follicle. Changes in P-bodies were determined by a subjective measurement. The numbers of dissected animals and replicates are described in S2 Table.
